# Lessons learned implementing and managing the DIVERT-CARE trial: practice recommendations for a community-based chronic disease self-management model

**DOI:** 10.1186/s12877-021-02248-0

**Published:** 2021-05-11

**Authors:** Darly Dash, Connie Schumacher, Aaron Jones, Andrew P. Costa

**Affiliations:** 1 Department of Health Research Methods, Evidence, and Impact, McMaster University, 1280 Main St. W, Hamilton, ON L8S 4L8 Canada; 2 Hamilton Niagara Haldimand Brant Local Health Integration Network, Hamilton, Canada; 3 Department of Nursing, Brock University, St. Catharines, Canada; 4 Schlegel Chair in Clinical Epidemiology & Aging, Schlegel-UW Research Institute for Aging, Waterloo, Canada

**Keywords:** Chronic disease management, Self-management, Home care, Implementation, Geriatric models of care

## Abstract

**Background:**

Chronic disease management models of care provide an opportunity to assist home care clients to manage their disease burden. However, pragmatic trial management practices and lessons learned from such models are poorly illustrated in the literature.

**Methods:**

We describe the processes of implementing a community-based cardiorespiratory self-management model, known as DIVERT-CARE, across the home care programs of three health regions in Canada. The DIVERT-CARE model is a multi-component complex intervention that identifies home care clients at the highest risk of deterioration and provides them with resources and capacity to manage their conditions. We conducted a retrospective analysis of baseline participant characteristics, needs assessments, reviewed findings from site visits and a national workshop with study partners, and examined other study documentation.

**Results:**

Three home care regions in Canada participated in the study. A robust and data-driven review of each site was necessary to understand the local context, home care caseloads, structure of local systems, and intensity of resources, which influenced study processes. The creation of an intervention framework highlighted the need to adapt the intervention in a way that was sensitive to the local context while maintaining intervention outcomes.

**Conclusion:**

Our detailed review showcases the relevant activities and on-the-ground steps needed to manage and conduct a multi-site pragmatic trial in home care. This example can help other researchers in implementing multi-disciplinary and multi-component care models for practice-based research.

**Supplementary Information:**

The online version contains supplementary material available at 10.1186/s12877-021-02248-0.

## Background

Implementing chronic disease management (CDM) models of care to support home care clients can reduce the burden of disease, provide access to resources and capacity to manage conditions, and be a cost-effective way of meeting the complex needs of older adults [1, 2]. However, rarely are management practices and lessons learned of CDM trials shared for the benefit of others [3–5]. Research on CDM models does not provide enough detail on implementation in terms of program design, necessary adaptations and strategies, and personnel and team involvement [6]. Without knowledge of practical implementation strategies, others may find it challenging to apply findings to daily practice which impacts the spread and sustainability of interventions and models of care [6].

CDM is the ongoing care and support to those living with chronic disease by providing the skills, resources, and knowledge for daily self-management. Research shows that CDM strategies improve home care client outcomes and reduce unnecessary hospitalizations [7–13]. The Chronic Care Model, developed by Wagner et al., lists essential components for high-quality CDM: a proactive multi-disciplinary team, effective self-management support, collaborative care plans, informed and activated clients, access to health system resources in the community, use of clinical information systems for communication and evaluation, delivery system design, and decision support [14].

Home care clients are heterogeneous in their medical needs, are medically complex with very high rates of visiting the emergency department (ED), and have poor access to chronic disease management within the home [15–18]. Most live at home with varying degrees of caregiving support [19], and over 2 million in Canada receive home care services provided by trained staff [20]. Nurses figure prominently in the management of home care clients, however, nursing services are often contracted with multiple agencies and tend to focus on reactive, problem-based measures, rather than preventative, health-promotion based care [21]. These ongoing challenges and gaps in care for home care clients can be addressed by developing a home-based comprehensive chronic disease self-management program.

Targeting home care clients most in need of CDM support is an efficient use of health resources. The Detection of Indicators and Vulnerabilities for Emergency Room Trips (DIVERT) scale is a validated prognostic case-finding tool used to identify home care clients with cardiorespiratory symptoms and conditions [22]. In accordance with the DIVERT scale score, we created the DIVERT-CARE intervention, which was designed through a scientific panel with clinical experts and a review of relevant clinical guidelines [23]. It is a complex self-management intervention targeting home care clients with cardiorespiratory symptoms at risk of a future ED visit. We previously tested DIVERT-CARE in a pilot study in a non-randomized cluster trial in Southwestern Ontario, Canada, with positive results showcasing its effectiveness [24]. DIVERT-CARE is currently undergoing further testing through a pan-Canadian pragmatic, cluster-randomized, multi-centre trial (#NCT 03012256) [25]. We have recruited and followed participants while collecting data for evaluation. Further details regarding the trial protocol are published elsewhere [25] and as part of a large program of research, multiple publications are anticipated.

This paper describes processes and considerations while implementing a nurse-led complex CDM model of care in home care programs of three health regions in Canada. We describe the steps taken to implement a real-world trial with moving systems, structures, people, and processes. Lessons learned are shared to support others in anticipating potential problems and identifying solutions for complex models of care.

## Methods

### Overview of the DIVERT-CARE model

A growing number of healthcare systems work with home care populations to address care needs pre-emptively before health deteriorates necessitating an ED or hospital visit. DIVERT-CARE is a model developed to use existing clinical guidelines and education resources/tools to promote self-management in the home care system [23]. The model was updated and evolved from the initial pilot testing in Ontario in 2014, where the absolute risk of an ED visit was reduced by 20% over the 7-month follow-up [24]. The theoretical foundations of DIVERT-CARE are from the Chronic Care Model [26], the population-based care approach [27], and principles of person-centred care [28]. We outline the principles of DIVERT-CARE in Table 1. DIVERT-CARE made use of a multi-disciplinary approach involving nurses, nurse practitioners, care coordinators of various professional designations, pharmacists, physicians, and social workers. The home care staff that delivered the intervention participated in a comprehensive training program and were able to identify and coordinate services towards clients.

**Table 1:** Principles of DIVERT-CARE

	Principles	Steps taken for Population-Based Care Approach
1.	Multi-disciplinary teams at each site are trained on the protocols and resources related to each cardio-respiratory management model component.	Baseline analytics were conducted to understand clients’ needs and preferences who were identified by the DIVERT scale score. This provided context for the resources and how they needed to be modified.
2.	Teams identify steps required to deliver the intervention(s)	Analytics were discussed at the operation level to understand the impact on intervention delivery. Regional implementation teams provided information on their health system and resources. In-person exercises were facilitated, and projections were conducted to identify human resource levels needed to deliver the intervention.
3.	Deployment of the nurse-led cardio-respiratory management model that engages clients, families, and caregivers to ensure that adequate resources are dedicated to supporting the interventions across the intervention caseloads while ensuring long-term sustainability	Supported by the national implementation team, regional implementation teams used identified steps and resources to deliver CDM. Ongoing virtual and in-person support occurred throughout the trial period to return to earlier steps to address challenges, provide training for additional personnel, and for ongoing enhancement of processes.

**Fig. 1:**
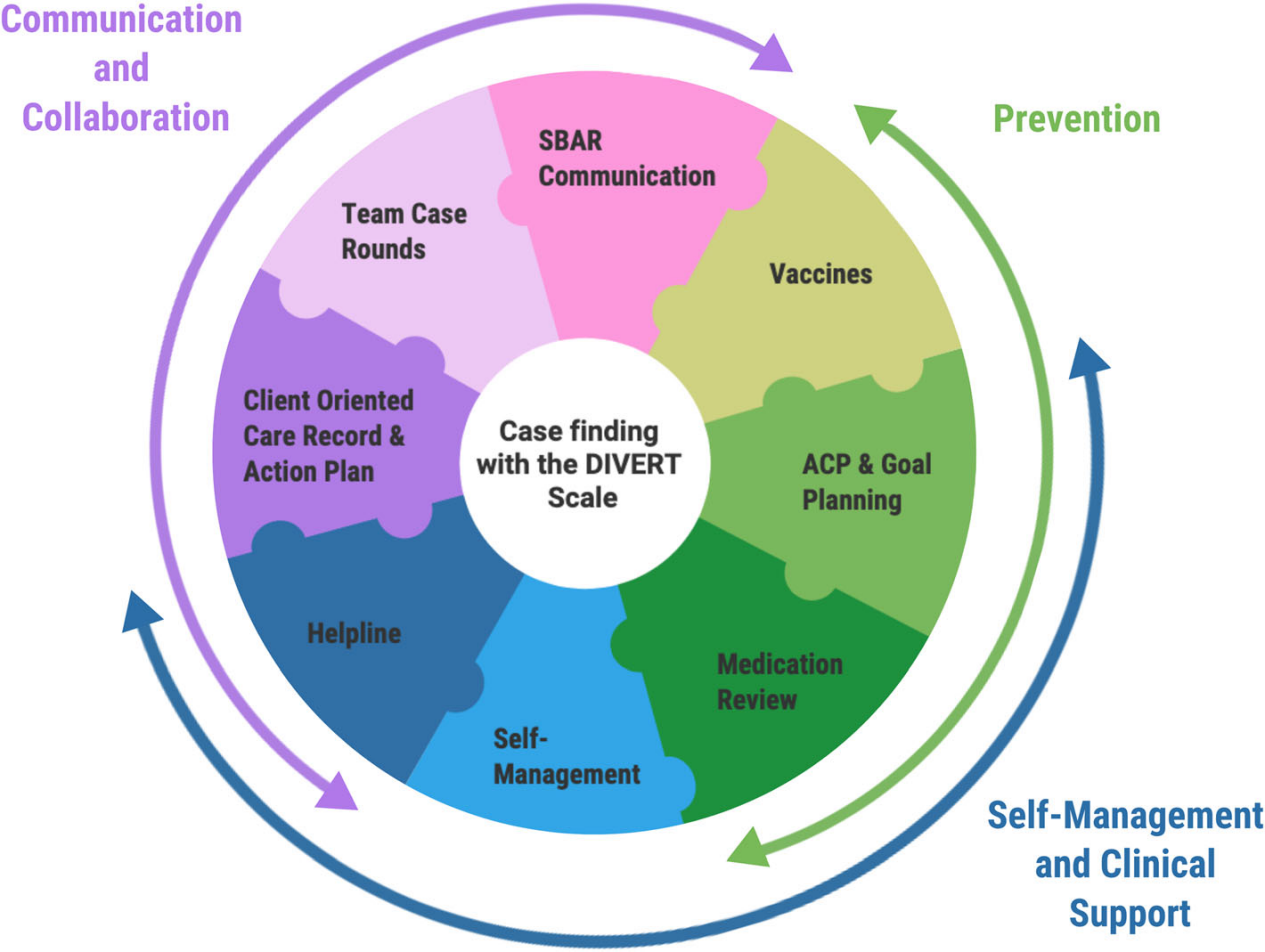
DIVERT-CARE pillars and components

The interRAI Home Care Assessment (interRAI-HC) and the Resident Assessment Instrument – Home Care (RAI-HC), are standardized assessment tools used to guide care and service planning for home care clients in community settings across the world [29, 30]. The interRAI-HC or RAI-HC has several embedded scales and algorithms derived from the assessment items, which are externally validated and used for decision support on health service needs [30]. They are completed by Care Coordinators or Case Managers for all long-stay home care clients. The interRAI-HC or RAI-HC automatically generates the DIVERT Scale score which identifies clients with cardiorespiratory symptoms at-risk of a future ED visit [22]. The comprehensive intervention was then targeted towards these risk factors and implemented by the home care services team in each regional program. Figure 1 depicts the components of DIVERT-CARE**.** The components are delivered over 15-weeks by trained staff, with the home care client’s consent for any or all components. Each component in the model has a specific function; however, the component’s delivery may be adapted. Further details on the comprehensive model and its foundations are reported in a previous publication [23].

### Intervention settings

Home care programs from distinct health regions in three Canadian provinces planned for implementation of the DIVERT-CARE intervention. Each region represents a different geographical and health system context within a publicly funded governance. These regions included Hamilton Niagara Haldimand Brant Local Health Integration Network (HNHB LHIN) in Ontario, Western Health (WH) in Newfoundland and Labrador, and Vancouver Island Health Authority (VIHA) in British Columbia.

### Coordination and management structure

Overall coordination and management of the DIVERT-CARE trial occurred through McMaster University, Ontario by the national implementation team. The national implementation team included the Study Principal Investigator, the Clinical Lead Coordinator responsible for overall trial management, implementation and evaluation support, and a data analyst, and no changes to this team occurred over trial implementation. The Clinical Lead Coordinator was, by profession, a registered nurse with over 20 years of experience in EDs and home care service provision, and has specifically focused on the management of chronic diseases.

The regional implementation teams included Site Principal Investigators who oversaw the project in their jurisdiction, Site Operational Leads responsible for intervention training and regional implementation, and frontline home care personnel for intervention delivery. The professional background of the leadership roles of Site Principal Investigators and Site Operational Leads included registered nursing or occupational therapy. All site-level personnel brought various strengths to the study due to experience with clinical education, information management, and health data quality.

### Review of project implementation processes

We retrospectively reviewed activities from intervention design, implementation, to completion of the study to understand processes and lessons learned. Data sources for this review included: internal documentation on the intervention, training materials, meeting agendas and notes based on ongoing engagement, results from needs assessments, findings from iterative site visits, outputs and notes from a national workshop with study partners, and data from each health region on their home care client population to understand regional-specific data. The results that follow highlight key areas of focus for implementing complex models of care, based on our detailed program review.

## Results

Enrollment into the DIVERT-CARE Trial occurred from February 2018 to the final six-month data follow-up in May 2020. We enrolled approximately 899 clients across the three sites, with initial targets of 1100 clients [25].

Our team engaged with each site before, during, and after trial implementation by working collaboratively to make the model scalable and adaptable to specific organizational and system practices. Over the course of the study, the Clinical Lead coordinator and Site Operational Leads met virtually every 2 weeks to discuss the intervention, enrollment, and implementation issues. A common issue discussed during these meetings was turnover in personnel, which was addressed by additional hiring and training of frontline personnel through in-person and virtual training. Beyond regular meetings, site visits were conducted by the Study Principal Investigator and/or the Clinical Lead Coordinator to meet with program leads, frontline personnel, and specialty groups to build capacity and assess education and training needs. These site visits established vital collaborations between the national and regional implementation teams but also facilitated new connections across local health teams that do not normally work together. For example, a telemonitoring medicine program worked together with home care personnel to deliver part of the intervention. Additional site visits over the study served to support local leads, observe team rounds, assist with the enrollment process, interact with intervention personnel, and provide motivation and encouragement. Overall, the early engagement allowed the national implementation team to consider pre-implementation issues to pre-emptively adapt DIVERT-CARE, and ongoing collaborations allowed for addressing concerns as they arose.

### Context of participating home care programs

Site visits, collaborations, and a needs assessment helped to understand the environment of each site. We describe the local context of each home care program below in order to understand the structure, intensity of available resources, and home care caseloads.

#### Hamilton Niagara Haldimand Brant Local Health Integration Network (HNHB LHIN)

The HNHB LHIN is one of 14 LHINs located in the province of Ontario. HNHB provides home and community care services for a diverse urban and rural population of 1.4 million people over a geographical area of approximately 6600 km^2^. Care Coordinators complete the interRAI HC assessment with clients and are healthcare professionals with the designations of Registered Nurse, Occupational Therapist, Physiotherapist, Dieticians, and Social Workers. The LHIN contracts care out to service providers and an internal clinical team comprised of registered nurses, nurse practitioners, and respiratory therapists to complex clients. Each Care Coordinator maintains a caseload of approximately 125 clients. Caseloads may be geographically clustered or aligned with primary care physician attachment. In the DIVERT-CARE study, HNHB had 16 caseloads enrolled (6 intervention, 10 control) from the Niagara and Haldimand-Norfolk sub-regions.

#### Western Health (WH)

WH is one of four regional health authorities located in the province of Newfoundland and Labrador. WH provides health services to a primarily rural population of approximately 77,720 people. Clients are assessed by a Case Manager using the RAI-HC assessment tool. Case Managers are health professionals with the designation of Registered Nurse or Registered Social Worker. The Case Manager assesses the client and arranges community and home support services. Each Case Manager maintains a caseload of approximately 80–90 clients, and caseloads are geographically based. All of WH was involved in the DIVERT-CARE study with 37 caseloads (13 intervention, 24 control).

#### Vancouver Island Health Authority (VIHA)

VIHA is one of six health authorities in the province of British Columbia. VIHA provides healthcare to over 785,500 people across a geographic area of approximately 56,000 km^2^. Clients are assessed by a Case Manager using the RAI-HC assessment tool. Case Managers are health professionals from various disciplines, including Registered Nurse, Occupational Therapist, Physiotherapist, or Social Worker. Case Managers work within a neighbourhood model with interdisciplinary teams to coordinate access and deliver community health services. In this region, home health monitoring is used to monitor clients living with conditions such as heart failure and chronic obstructive pulmonary disease. Each Case Manager is responsible for a caseload of approximately 80–90 persons. Within VIHA, the Nanaimo sub-region partook in the DIVERT-CARE study with 13 caseloads (4 intervention, 9 control).

### Training of intervention personnel

For intervention personnel training, an orientation with training materials was designed and shared with each participating home care program [31]. Physical materials were delivered to sites and other materials were shared electronically. The materials provided structure and content so that each program could implement the components of the DIVERT-CARE model. Each site used the materials uniquely to arrange for intervention implementation. In HNHB and WH, training was delivered by one facilitator (the Clinical Lead Coordinator) with an in-person presentation using the same format and materials. However, due to site preference, training in VIHA was conducted by an internal educator part of the health authority who used the trial materials to disseminate training through a self-directed e-learning platform as well as in-person instruction.

### Client characteristics

Data from each home care program was obtained to understand the caseloads and client characteristics unique to the local region. Through this data-driven process, we understood demographic variables, health characteristics, and chronic conditions in order to target intervention resources appropriately. Table 2 describes the baseline characteristics of home care clients in each healthcare region.

**Table 2:** Baseline characteristics of eligible home care clients across trial sites, January–December 2016

Home Care Client Characteristics	HNHB	WH	VIHA
	***n*** = 7709	***n*** = 375	***n*** = 1767
**Demographics**			
Age, yr (Median (Q1, Q3))	83 (74–89)	80 (71–86)	86 (79–90)
Sex, male	39.9%	37.6%	39.2%
Lived alone	37.0%	44.9%	47.9%
Lived alone w/ impaired cognition	22.7%	17.6%	35.4%
**Health**			
ADL Impairment^a^			
Independent/Supervision	50.0%	51.2%	61.8%
Limited/Extensive	38.5%	38.4%	30.0%
Maximal/ Dependent	11.5%	10.4%	8.3%
Cognitive Impairment^b^			
Intact / Borderline intact	51.7%	71.2%	34.8%
Mild / Moderate	44.5%	25.9%	59.0%
Severe	3.8%	2.9%	6.3%
Number of Medications			
0–4	8.0%	3.2%	10.1%
5–8	26.4%	31.7%	33.2%
9 or more	65.6%	65.1%	56.6%
Any mood symptom	33.5%	29.1%	34.4%
Bladder incontinence	44.4%	36.8%	41.2%
Fall in last 90 days	51.9%	36.3%	42.6%
**DIVERT Subgroups**^**c**^			
9	14.8%	25.1%	19.6%
10	11.2%	22.1%	19.1%
14	42.5%	34.9%	31.4%
15	31.4%	17.9%	29.9%
**Chronic Conditions**			
Congestive heart failure	25.2%	29.3%	35.7%
Stroke	21.9%	14.7%	22.8%
Hypertension	71.8%	77.3%	67.4%
Chronic obstructive pulmonary disease	29.0%	40.3%	32.6%
Diabetes	31.6%	40.3%	25.0%
Dementia	19.3%	8.3%	35.8%

Note: *ADL* Activities of daily living, *Q1* Quartile 1, *Q3* Quartile 3

^a^ADL Hierarchy Scale: Includes personal hygiene, locomotion, eating and toileting

^b^Cognitive performance scale, which measures the degree of cognitive impairment

^c^DIVERT measures risk of future ED utilization

HNHB was the first region in the DIVERT-CARE study so the intervention and its components were initially designed for HNHB’s home care program and client characteristics. However, based on data in Table 2, we saw lower levels of dementia in the WH population which was promising for the self-management component and possible client education retention. Further, Table 2 indicates that in VIHA, there are a higher proportion of clients with mild to moderate cognitive impairment and higher rates of dementia. These individuals were also more likely to live alone. Given that the DIVERT-CARE intervention included a self-management component with client education, we used this information to consider how to deliver the support best to clients, such as using telemonitoring support in VIHA. Information gathered from the baseline characteristics in Table 2 informed adaptations that worked with local structures and resources.

### Intervention framework and site adaptations

We conducted a national in-person one-day workshop with representation from the national and regional implementation teams. Members included nursing leaders, regional trial leads, and implementation and research methods experts. The objective of the workshop was to gain an understanding of each regional setting, develop the overall intervention theory and logic model, outline necessary site-based adaptations, and create process management flows.

**Fig. 2:**
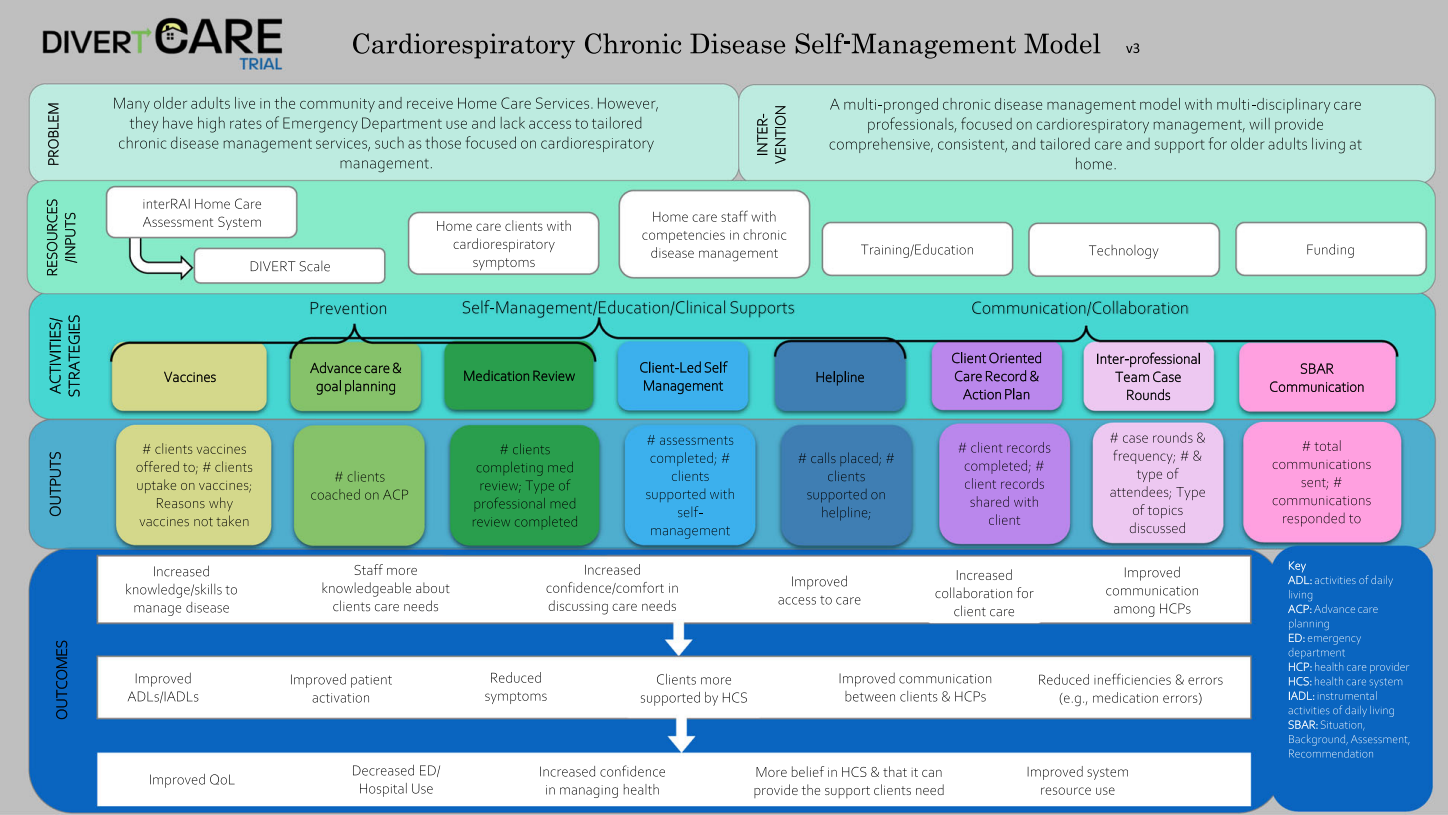
DIVERT-CARE framework

Each site described in detail how their home care programs operated, client characteristics, and resources available. All workshop members then worked together to co-create an intervention logic model identifying the core components of DIVERT-CARE. Figure 2 depicts the overall logic model or framework of DIVERT-CARE, as conceptualized during the national workshop. Each home care program implemented all main components of DIVERT-CARE.

Through the process of creating the logic model, we recognized that we could not be completely uniform across programs due to regional variation in context, process, and resources. Workshop members were further divided into teams to consider the desired outcomes and what adaptations to resources and structures were needed to best meet targets. As a result of our workshop, the purpose of each component of DIVERT-CARE was standardized, while the actual form or operationalization of the component was allowed to vary according to local needs and resources [32]. Additional file 1 depicts the adaptions to various components of DIVERT-CARE for the three participating home care programs. The main reasons for adaptations were differences in the healthcare system infrastructure and resources, characteristics of the home care client population receiving services (Table 2), and geographic characteristics.

**Fig. 3:**
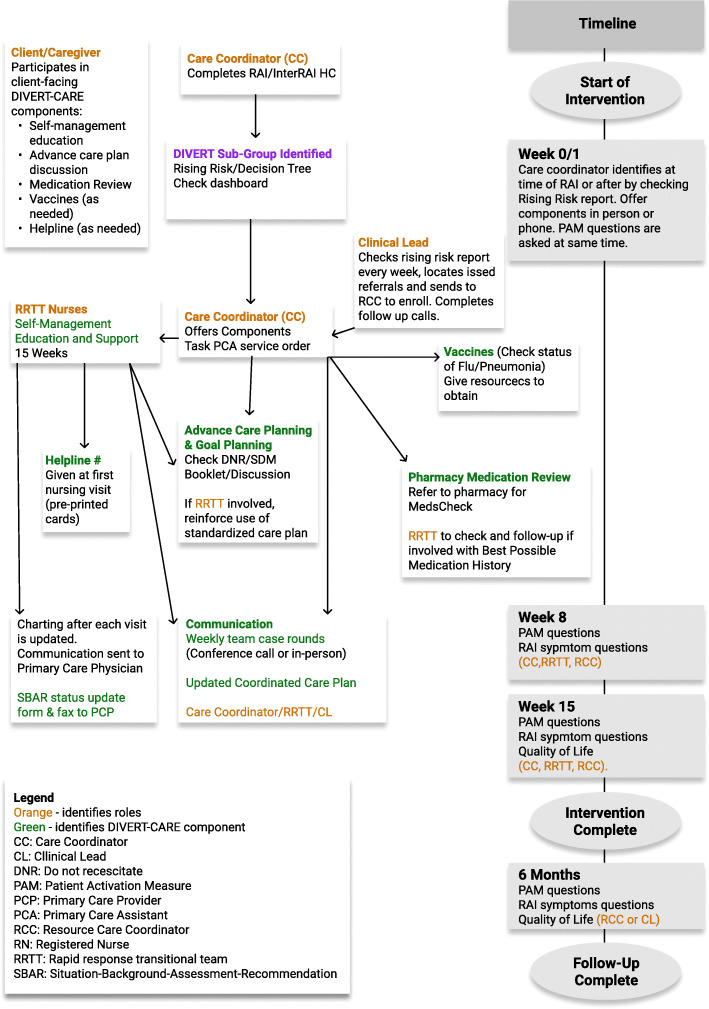
DIVERT-CARE HNHB process pathway

Lastly, each regional team created a process pathway for how the trial would be implemented within their home care program. The process supported the national implementation team in visualizing and understanding the human resources needed, and the sequence of implementation activities. Figure 3 displays the process pathway for one site.

## Discussion

To our knowledge, we are the first to highlight the real groundwork for implementing a pragmatic trial in the complicated home care system. The DIVERT-CARE model is currently the largest pragmatic home care trial in Canada, and our team will share results in a forthcoming publication. Pragmatic trials are needed for health systems to adopt evidence-based interventions in routine practical settings [33]. Practice change involving complex interventions does not occur automatically and requires intentional strategic planning [34, 35]. Other countries or jurisdictions may learn from our trial management and implementation processes, particularly how healthcare interventions operating in everyday settings can still meet the needs of the clients they serve. Based on our experiences implementing DIVERT-CARE, we highlight several lessons learned that researchers need to consider for future trial implementation research in home care.

### Intensity of human resources and time: overcome the wall of implementation fatigue

Trials, by nature, are human resource and time-intensive at all levels. In our study, enrollment and follow-up were planned around available human resources in each health region, with enrollment staggered according to caseload size. This ramp-up period allowed for testing and tailoring of the study processes. We recommend that trial managers provide frontline personnel with sufficient time for learning new processes as they embed practice change.

Our study had both management and frontline personnel turnover, which we addressed throughout with ongoing communication, training, and site visits. Due to our engagement and mitigation strategies, each regional implementation team still retained knowledge and control. Ongoing training has been previously reported as a facilitator of uptake and buy-in [34]. We recommend that trial managers proactively create contingency plans for personnel attrition and include time for ongoing training in the implementation strategy. A recent Canadian study found that staffing gaps contribute to at least 10% loss in recruitment time and there was a need to train at least one-third more personnel than initially allocated [36].

### Invest in readiness assessments to better understand the contextual issues at the site

Our trial involved an extensive understanding of the local system and leveraged population-level data to understand client characteristics, which informed project resource planning. This allowed for effective targeting and deployment of a real-world intervention based on client needs. We recommend conducting a robust readiness assessment of sites. Other implementation experts have found utility in assessing sites for readiness including understanding capacity and process measure data [35–37].

### Be flexible and open to changes that support the intervention in the local area

In our trial, we used the findings from the readiness assessment to consider informed adaptations that reflect the original intent and outcomes of the model, without being completely uniform in the delivery of the intervention [38, 39]. Backwards mapping allowed us to focus on reaching the intended outcome which is a strategy used in bottom-up approaches to implementation [40]. This process may further enhance the uptake and sustainability of complex models of care beyond the trial period [41, 42]. We recommend that others designing complex models of care focus on the methods that allow them to best reach intended outcomes for local sustainability.

### Establish local teams with rapport and influence who are proximal to day-to-day practice

Those involved in trials should consider if teams and departments are siloed or work together, and who has the authority to ensure the ongoing delivery of the intervention. In our study, we aimed to understand human resource structure, capacity, and chain of command when selecting regional implementation teams as this can affect the degree of control over study procedures. We also established regular communication channels and made new connections amongst stakeholders that did not previously work together. We recommend that trial managers facilitate collaborations beyond the management team to build system capacity. Regular, personalized communication has been found to be time consuming but essential in successful trials [4, 36].

Our recommendations in this paper are based on a pragmatic trial for practice change in the home care system. The retrospective analysis of our trial and implementation processes was not originally conceptualized but provides important steps that may be generalizable. Our approach is unique as we describe the management, implementation processes, and lessons learned of a multi-site home care trial across Canada. Overall, the actions we have taken and lessons learned can inform trialists in the implementation of complex multi-disciplinary and multi-component care in a realistic and scalable way. Given that our team relied on core infrastructure and approaches from the initial stages, the probability and ease of further spread is likely [38, 43]. New work in the field should examine facilitators and barriers to trial implementation, and how they impact the sustainability of complex models of care.

## Conclusions

We show how the DIVERT-CARE model was implemented and adapted to work with home care programs involved in the DIVERT-CARE intervention. Adapted components in the model were co-created and rooted within each local system and the teams who implemented it. This practice not only facilitates implementation to the local context but provides lessons learned that can be referred to for an explanation of differences in trial outcomes between participating sites. This paper can be used as an implementation and trial process management exemplar to conduct practice-based research that guides and informs complex system change.

## Supplementary Information

**Additional file 1.** DIVERT-CARE components and associated adaptations. This table depicts each component of the DIVERT-CARE model and how each component was adapted for the three participating sites.

## Data Availability

The datasets used and/or analyzed during the current study are available from the corresponding author on reasonable request.

## Abbreviations

CDM: Chronic Disease Management
DIVERT: Detection of Indicators and Vulnerabilities for Emergency Room Trips
ED: Emergency Department
HNHB LHIN: Hamilton Niagara Haldimand Brant Local Health Integration Network
interRAI HC: interrai Home Care Assessment
RAI-HC: Resident Assessment Instrument – Home Care
VIHA: Vancouver Island Health Authority
WH: Western Health
