# Supplementary material for: Lessons learned implementing and managing the DIVERT-CARE trial: practice recommendations for a community-based chronic disease self-management model
Source: BMC Geriatr. 2021 May 11;21:303. doi: 10.1186/s12877-021-02248-0 (PMC8111935; doi:10.1186/s12877-021-02248-0)
Supplement: Supplementary file 1 — Additional file 1. DIVERT-CARE components and associated adaptations. This table depicts each component of the DIVERT-CARE model and how each component was adapted for the three participating sites. [file 12877_2021_2248_MOESM1_ESM.docx]

**Additional File 1.** DIVERT-CARE components and associated adaptations

| **DIVERT-CARE Intervention Components** | **Description** | **Adaptations to DIVERT-CARE sites** | | |
| --- | --- | --- | --- | --- |
|  |  | **HNHB** | **Western Health** | **VIHA** |
| **Client-Centered Self-Management** | In-home assessment and identification of DIVERT Scale Score by Care Coordinators/Case Managers  Nurses provided practical education and skills training to recognize and manage symptoms. | Care Coordinators complete the in-home assessment with the client. The existing Rapid Response Nursing program delivered the self-management education component. Standard practice includes completion of disease-specific education, self-management supports, and a Best Possible Medication History, and was in-line with requirements of DIVERT-CARE. | Use of existing Case Managers with a Registered Nursing designation, functioned in an adapted dual role to complete the assessment and deliver the self-management education. These Case Managers received training and education to deliver the component, which changed the role and frequency of contact with the client. The adaptation was in the delivery of this component. | Case Managers completed the in-home assessment. Clients received support through pre-existing programs in the region, the Home Health Monitoring Program, or Living Well with COPD/Heart Failure Health and Wellness Program. Registered Nurses or Nurse Clinicians delivered HF education, and COPD education was delivered by Respiratory Therapists. The adaptation was in the delivery of the component. |
| **Access to an immediate staffed helpline** | Helpline available to clients involved in the DIVERT-CARE Intervention to aid with self-management and problem resolution. | Dedicated phone line staffed by nurses involved in the intervention to support clients. Day hours only. | Dedicated phone line staffed by nurses involved in the intervention to support clients. 24-hour coverage. | Use of provincial staffed helpline to support clients, rather than at the site level. Provincial helpline is staffed by a qualified health service navigator. |
| **Promotion of Vaccines** | The following vaccines are offered and/or recommended for follow up with their primary care physician:   - Seasonal flu vaccine - pneumococcal polysaccharide (Pneu-P-23) | No change | No change | No change |
| **Advance Care and Goal Planning** | Consultation for advance care and goals of care planning, advanced care decisions, and communication of care wishes. | No change | No change | No change |
| **Medication Review** | Review of medication for safety, efficacy and appropriate use of medications, delivery options, and dispensing/packaging. | Use of a provincially funded program that provides comprehensive medication review at the pharmacy and in home. Home-bound clients had a contracted pharmacist visit them in the home. Lastly, clients who accepted self-management had a Rapid Response Nurse complete a Best Possible Medication History with them. | Medication reviews are not funded across the province within pharmacies. Instead, a partnership was created with local pharmacists and the school of pharmacy to adapt the delivery of this component. A designated pharmacist was secured to execute this component. | A dedicated pharmacist conducted medication reviews for complex clients. All their clients had the Best Possible Medication Review completed by their Case Manager during self-management. |
| **Interprofessional Team Case Rounds** | Regular care team meetings to discuss care plan, update goals, and how to support changing care needs. | Weekly team case rounds | Weekly team case rounds. Clients were included in interdisciplinary rounds creating a person-centered approach to ongoing care planning. A primary care centre was enlisted for clients without access to a physician. | Bi-weekly team case rounds were conducted due to:   1. wide geographical area 2. to accommodate existing meeting structures |
| **SBAR Communication** | SBAR formatted communication to convey relevant information and care updates to primary and specialist care providers. | No Change | No Change | No Change |
| **Client Oriented Care Record & Action Plan** | A succinct document entitled the “My Care Plan” to support continuity of care throughout the health system. Shared record of goals, plan of care, and community supports. | Use of the existing “Coordinated Care Plan” from the Ontario Ministry of Health and Long-term Care, ensuring consistency. The Coordinated Care Plan includes all components from the “My Care Plan” as designed for DIVERT-CARE. | Use of “My Care Plan” as designed for DIVERT-CARE. | Use of a care plan from VIHA, that was consistent with the content from “My Care Plan” as designed for DIVERT-CARE. |
